# Effect of high-dose polymeric nanoparticle micellar paclitaxel on improved progression-free survival in patients with optimally resected stage III or IV high-grade carcinoma of the ovary: a prospective cohort study with historical controls

**DOI:** 10.3389/fonc.2024.1203129

**Published:** 2024-02-09

**Authors:** Soo Jin Park, Joo-Hyuk Son, Tae-Wook Kong, Suk-Joon Chang, Hee Seung Kim

**Affiliations:** ^1^ Department of Obstetrics and Gynecology, Seoul National University Hospital, Seoul, Republic of Korea; ^2^ Division of Gynecologic Oncology, Department of Obstetrics and Gynecology, Ajou University School of Medicine, Suwon, Republic of Korea; ^3^ Department of Obstetrics and Gynecology, Seoul National University College of Medicine, Seoul, Republic of Korea

**Keywords:** polymeric nanoparticle micellar paclitaxel, bevacizumab, high-grade serous ovarian cancer, optimal debulking surgery, survival

## Abstract

**Introduction:**

We evaluated the effect of high-dose polymeric nanoparticle micellar paclitaxel (PM-Pac) on survival in patients with stage III-IV high-grade serous ovarian cancer (HGSC) who underwent upfront surgery.

**Methods:**

We prospectively recruited the patients who received PM-Pac (280 mg/m^2^) and carboplatin at an area under the curve (AUC) of 5 (cohort 1) in two tertiary centers between October 2015 and June 2019. As historical controls, we retrospectively collected data on those who received paclitaxel (175 mg/m^2^) and carboplatin (AUC 5; cohort 2) or paclitaxel (175 mg/m^2^), carboplatin (AUC 5) and bevacizumab (15 mg/kg; cohort 3).

**Results:**

A total of 128 patients were divided into cohorts 1 (n=49, 38.3%), 2 (n=53, 41.4%), and 3 (n=26, 20.3%). Cohort 1 showed better progression-free survival (PFS) than cohort 2 in all patients and those treated with optimal debulking surgery (ODS; median, 35.5 vs. 28.1 and 35.5 vs. 28.9 months; p ≤ 0.01) despite no difference in PFS between cohorts 1 and 3 and between cohorts 2 and 3. In particular, stage III disease was a favorable factor for PFS, whereas cohort 2 was related to worse PFS (adjusted hazard ratios, 0.456 and 1.834; 95% confidence interval, 0.263 – 0.790 and 1.061 – 3.171), showing no difference in PFS between cohorts 1 and 3 in those treated with ODS.

**Conclusion:**

High-dose PM-Pac improved PFS compared to conventional chemotherapy, and the change of paclitaxel to PM-Pac had as much effect on PFS as the addition of bevacizumab in patients with stage III-IV HGSC who underwent ODS.

## 1 Introduction

Paclitaxel combined with platinum such as cisplatin or carboplatin has become the standard medical treatment after upfront surgery for advanced ovarian cancer, which shows overall response rates of 73-75%, progression-free survival (PFS) of 18-19.4 months, and overall survival (OS) of 38-48.7 months (1, 2). Considering its cytotoxic effect, paclitaxel is still used as adjuvant therapy in the era of targeted or immune oncologic therapy (3–6).

To increase the solubility of paclitaxel, a fat-soluble anti-cancer agent, it is prepared with a micelle-forming vehicle, polyoxyl-35-castor oil (Cremophor EL) (7). However, Cremophor EL causes hypersensitivity reactions and neurotoxicity (8, 9), and impedes drug metabolism and tissue distribution because of non-linear pharmacokinetics (10). Even though Cremophor EL-free paclitaxel exhibits linear pharmacokinetics, it shows low biological responses because the altered pharmacokinetics and relevant toxicities are further increased when the dose is raised to increase activity (11).

To reduce dose-limiting toxicities (DLTs) and maximize the therapeutic effect in different types of cancers, polymeric nanoparticle micellar paclitaxel (PM-Pac) has been developed as a Cremophor EL-free formulation (12–14). Especially, phase I trials showed that the maximum tolerated dose (MTD) of PM-Pac was 390 mg/m**^2^** as monotherapy (15), and 260 mg/m**^2^** when combined with carboplatin (16), suggesting that PM-Pac may be safely used at higher doses than paclitaxel in patients with ovarian cancer. Moreover, PM-Pac/carboplatin reportedly showed a non-inferior effect with tolerable toxicities compared with conventional paclitaxel and carboplatin in the patients (17).

However, there is no study to evaluate the effect of PM-Pac compared with conventional regimens or targeted therapy for patients with ovarian cancer where external variables, including the International Federation of Gynecology and Obstetrics (FIGO) stage, histology and degree of tumor resection are controlled. Thus, we conducted a prospective cohort study for only patients with stage III-IV high-grade serous carcinoma of the ovary (HGSC) who received PM-Pac/carboplatin after upfront surgery, and its effect was evaluated by comparing with paclitaxel/carboplatin and paclitaxel/carboplatin/bevacizumab for those treated during the same period.

## 2 Materials and methods

### 2.1 Study design

We performed a prospective cohort study for patients with stage III-IV HGSC who received PM-Pac/carboplatin after ODS between October 2015 and June 2019, and compared its effect with paclitaxel/carboplatin and paclitaxel/carboplatin/bevacizumab for those treated during the sample period in two tertiary centers. Institutional Review Boards of Seoul National University Hospital (No. 1508-121-697) and Ajou University Medical Center (No.MED-OBS-15-321) approved this study in advance. This study was registered at ClinicalTrial.gov (No. NCT05300828). For the prospective cohort study, we consecutively recruited patients who were aged 18 years or older; had Eastern Cooperative Oncology Group (ECOG) performance status of 0-2; had stage III-IV HGSC; received PM-Pac/carboplatin after upfront surgery; signed the approved informed consent form. On the other hand, we excluded patients who had non-HGSC; received neoadjuvant chemotherapy followed by interval debulking surgery; had other malignancies affecting the prognosis; and received other targeted or immune oncologic therapy except bevacizumab.

As historical controls, we retrospectively collected data on those who received paclitaxel/carboplatin or paclitaxel/carboplatin/bevacizumab during the same period. The eligibility criteria for the retrospective group were the same as those for the prospective group except for the chemotherapeutic regimen.

### 2.2 Treatment

After upfront surgery, all patients were divided into the following three groups; cohort 1, the prospective cohort where PM-Pac/carboplatin were used; cohort 2, one of the retrospective cohorts where paclitaxel/carboplatin were used; cohort 3, the other retrospective cohort where paclitaxel/carboplatin/bevacizumab were used. In cohort 1, we administered Genexol-PM (Samyang Co., Seoul, Republic of Korea) of 280 mg/m^2^ and carboplatin at an area under the curve (AUC) of 5 every three weeks, whereas paclitaxel of 175 mg/m^2^ and carboplatin at AUC 5.0 were used as the conventional chemotherapy every three weeks in cohort 2. Furthermore, bevacizumab of 15 mg/kg was administered with the conventional chemotherapy for six cycles, and then it was added for up to 12 more cycles as maintenance therapy in cohort 3.

### 2.3 Data collection

We collected clinicopathologic information about age, FIGO stage, BRCA mutation, degree of tumor resection, cycles of chemotherapy, tumor response, and survival. Optimal debulking surgery (ODS) was defined when the size of residual tumors was 1 cm or less. PFS was defined as the time interval from the date of surgery to the date of disease relapse or last follow-up, and OS was defined as the time interval from the date of surgery to the date of cancer-related death or last follow-up.

### 2.4 Statistical analysis

We compared variables among the three cohorts with analysis of variance, Kruskal-Wallis and chi-square tests, and survival variables were evaluated by the Kaplan-Meier methods with the log-rank test. Prognostic factors were determined using Cox’s proportional hazard regression analysis using hazard ratio (HR) and 95% confidence interval (CI). For statistical analysis, we used SPSS software version 22.0 (SPSS Inc., Chicago, IL, USA), and p <0.05 was considered statistically significant.

## 3 Results

### 3.1 Patient characteristics

Among a total of 128 patients included in this study, we consecutively recruited 49 (38.3%) treated with PM-Pac/carboplatin in cohort 1, whereas 53 (41.4%) and 26 (20.3%) were included in cohorts 2 and 3, respectively. **Table 1** depicts patients’ characteristics, and patients with stage IV disease were more in cohort 3 than in cohorts 1 and 2 (69.2% vs. 20.4% and 34%; p <0.05). However, there were no differences in age, BRCA status, degree of tumor resection, cycles of chemotherapy, and tumor response among the three cohorts.

**Table 1:** Patients’ characteristics.

Characteristics	Cohort 1 (n=49, %)	Cohort 2 (n=53, %)	Cohort 3 (n=26, %)	p value
Age (y)	55 (34, 74)	52 (33, 79)	49 (33, 75)	0.229
FIGO stage				<0.01
III	39 (79.6)	35 (66)	8 (30.8)	
IV	10 (20.4)	18 (34)	18 (69.2)	
BRCA status				0.462
BRCA 1 or 2 mutation	12 (27.3)	11 (27.5)	4 (15.4)	
Wild type or VUS	37 (72.7)	42 (72.5)	22 (84.6)	
Degree of tumor resection				0.023
Optimal	49 (100)	46 (86.8)	22 (84.6)	
Suboptimal	0 (0)	7 (13.2)	4 (15.4)	
Cycles of chemotherapy				0.371
<6	7 (14.3)	6 (13.2)	1 (0.8)	
≥6-	42 (85.7)	46 (86.8)	25 (96.2)	
Tumor response				0.187
Complete response	32 (65.3)	28 (52.8)	11 (42.3)	
Partial response	4 (8.2)	11 (20.8)	9 (34.6)	
Stable disease	12 (24.5)	12 (22.6)	5 (19.2)	
Progressive disease	1 (2)	2 (3.8)	1 (3.8)	

FIGO, International Federation of Gynecology and Obstetrics; VUS, variant of uncertain significance.

Cohort 1, polymeric micellar paclitaxel and carboplatin; cohort 2, paclitaxel and carboplatin; cohort 3, paclitaxel, carboplatin and bevacizumab.

### 3.2 Survival

**Figure 1:**
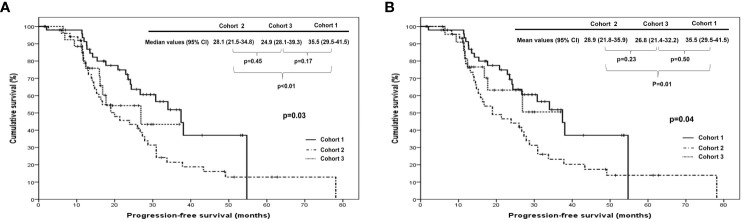
Comparison of progression-free survival among cohorts 1, 2, and 3 in **(A)** all patients and **(B)** patients who underwent optimal debulking surgery: cohort 1, polymeric micellar paclitaxel and carboplatin; cohort 2, paclitaxel and carboplatin; cohort 3, paclitaxel, carboplatin and bevacizumab.

**Figure 2:**
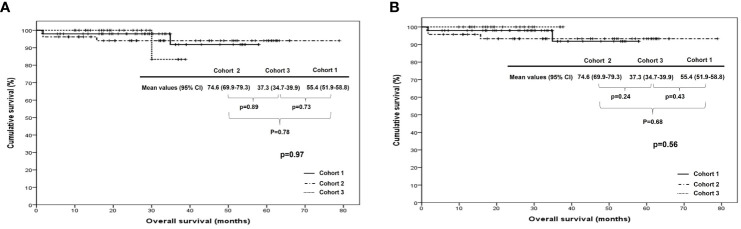
Comparison of overall survival among cohorts 1, 2, and 3 in **(A)** all patients and **(B)** patients who underwent optimal debulking surgery: cohort 1, polymeric micellar paclitaxel and carboplatin; cohort 2, paclitaxel and carboplatin; cohort 3, paclitaxel, carboplatin and bevacizumab.

When we compared PFS and OS among cohorts 1, 2, and 3 in all patients, cohort 1 showed better PFS than cohort 2 (median, 35.5 vs. 28.1 months; p <0.01), whereas there were no differences in it between cohorts 1 and 3 (median, 35.5 vs. 24.9 months; p=0.17) and between cohorts 2 and 3 (median, 28.1 vs. 24.9 months; p=0.45). In patients treated with ODS, cohort 1 also demonstrated better PFS than cohort 2 (median, 35.5 vs. 28.9 months; p=0.01) despite no differences in it between cohorts 2 and 3 (median, 26.8 vs. 28.9 months; p=0.23) and between cohorts 1 and 3 (median, 35.5 vs. 26.8 months; p=0.50; **Figure 1**). Nevertheless, OS was not different among the three cohorts in all patients and those treated with ODS (**Figure 2**).

### 3.3 Prognostic factors

**Figure 3:**
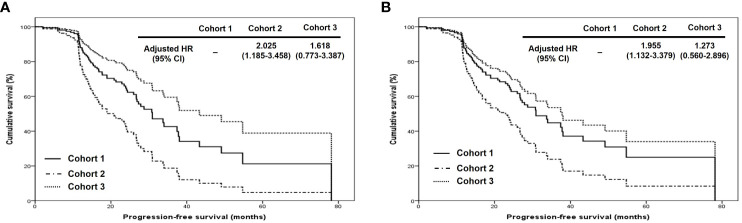
Comparison of progression-free survival proportion by Cox proportional hazards regression analysis among cohorts 1, 2, and 3 in **(A)** all patients and **(B)** patients who underwent optimal debulking surgery: cohort 1, polymeric micellar paclitaxel and carboplatin; cohort 2, paclitaxel and carboplatin; cohort 3, paclitaxel, carboplatin and bevacizumab (HR, hazard ratio).

**Figure 4:**
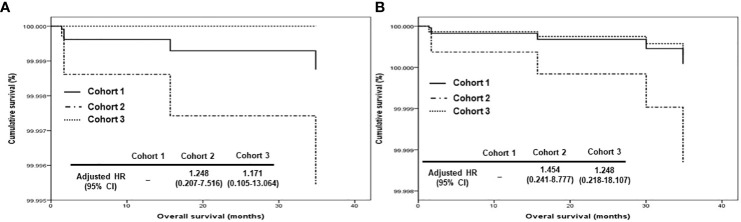
Comparison of overall survival proportion by Cox proportional hazards regression analysis among cohorts 1, 2, and 3 in **(A)** all patients and **(B)** patients who underwent optimal debulking surgery: cohort 1, polymeric micellar paclitaxel and carboplatin; cohort 2, paclitaxel and carboplatin; cohort 3, paclitaxel, carboplatin and bevacizumab (HR, hazard ratio).

In terms of PFS, stage III disease and ODS were favorable factors in all patients (adjusted HRs, 0.500 and 0.348; 95% CIs, 0.306 – 0.817 and 0.156 – 0.777). On the other hand, stage III disease was still a favorable factor (adjusted HR, 0.456; 95% CI, 0.263 – 0.790), whereas cohort 2 was related to worse PFS in those treated with OFS (adjusted HR, 1.834; 95% CI, 1.061 – 3.171; **Table 2**). Regarding OS, ODS was the only favorable factor in all patients (adjusted HR, 0.044; 95% CI, 0.008 – 0.248), whereas there was no factor affecting OS in those treated with ODS (**Table 3**). When we evaluated the hazards of disease progression and cancer-related death, cohort 3 tended to have a lower hazard than cohorts 1 and 3 in all patients, whereas cohorts 1 and 3 tended to have similar hazards, with less hazard than cohort 2 (**Figures 3**, **4**).

**Table 2:** Factors affecting progression-free survival.

Factors	HR	95% CI	p value	Adjusted HR	95% CI	p value
All patients						
Age <52 y	0.912	0.568 – 1.464	0.701	–	–	–
Stage III disease	0.530	0.326 – 0.862	0.011	0.500	0.306 – 0.817	0.006
BRCA mutation	1.043	0.614 – 1.773	0.875	–	–	–
Cycles of chemotherapy ≥6	0.892	0.358 – 2.222	0.807	–	–	–
ODS	0.398	0.180 – 0.879	0.023	0.348	0.156 – 0.777	0.010
Compared with cohort 1						
Cohort 2	2.025	1.185 – 3.458	0.010	–	–	–
Cohort 3	1.618	0.773 – 3.387	0.202	–	–	–
Patients treated with ODS						
Age <52 y	0.918	0.558 – 1.513	0.738	–	–	–
Stage III disease	0.512	0.307 – 0.854	0.010	0.456	0.263 – 0.790	0.005
BRCA mutation	1.033	0.584 – 1.827	0.912	–	–	–
Cycles of chemotherapy ≥6	1.410	0.441 – 4.507	0.562	–	–	–
Compared with cohort 1						
Cohort 2	1.955	1.132 – 3.379	0.016	1.834	1.061 – 3.171	0.030
Cohort 3	1.273	0.560 – 2.896	0.565	–	–	–

ODS, optimal debulking surgery.

Cohort 1, polymeric micellar paclitaxel and carboplatin; cohort 2, paclitaxel and carboplatin; cohort 3, paclitaxel, carboplatin and bevacizumab.

**Table 3:** Factors affecting overall survival.

Factors	HR	95% CI	p value	Adjusted HR	95% CI	p value
All patients						
Age <52 y	0.017	0.003 – 12.212	0.224	–	–	–
Stage III disease	2.512	0.293 – 21.520	0.401	–	–	–
BRCA mutation	0.033	0.012 – 14.520	0.412	–	–	–
Cycles of chemotherapy ≥6	0.044	0.008 – 0.248	<0.001	–	–	–
ODS	0.041	0.004 – 0.351	0.042	0.044	0.008 – 0.248	<0.001
Compared with cohort 1						
Cohort 2	1.248	0.207 – 7.516	0.809	–	–	–
Cohort 3	1.171	0.105 – 13.064	0.898	–	–	–
Patients treated with ODS						
Age <52 y	0.016	0.002 – 22.187	0.264	–	–	–
Stage III disease	2.144	0.240 – 019.182	0.495	–	–	–
BRCA mutation	0.034	0.008 – 31.637	0.468	–	–	–
Cycles of chemotherapy ≥6	0.050	0.008 – 0.301	0.001	–	–	–
Compared with cohort 1						
Cohort 2	1.454	0.241 – 8.777	0.683	–	–	–
Cohort 3	1.248	0.218 – 18.107	0.980	–	–	–

ODS, optimal debulking surgery.

Cohort 1, polymeric micellar paclitaxel and carboplatin; cohort 2, paclitaxel and carboplatin; cohort 3, paclitaxel, carboplatin and bevacizumab.

## 4 Discussion

This study showed the potential that PM-Pac/carboplatin might be superior to paclitaxel/carboplatin with a similar effect to paclitaxel/carboplatin/bevacizumab on improved PFS, in particular, for patients with stage III-IV HGSC who underwent ODS. These findings suggest that PM-Pac may be more beneficial than conventional paclitaxel in improving the prognosis in the patients.

In a previous randomized phase II trial, PM-Pac/carboplatin showed the only non-inferior effect on PFS with no benefit of high-dose paclitaxel by formulation change (17). However, early-stage disease, different types of histology and tumor differentiation, and various degrees of tumor resection would act as a bias for evaluating the effect of PM-Pac/carboplatin on survival compared with paclitaxel/carboplatin in the phase II trial. On the other hand, we adjusted these variables in this study and then found that the change from conventional paclitaxel to PM-Pac was comparable to the addition of bevacizumab for improving PFS in patients with advanced HSGC who underwent ODS.

Even though high-dose paclitaxel formulated conventionally demonstrated no benefit of tumor response and survival with more relevant toxicities in breast cancer (18), nanoparticle albumin-bound paclitaxel showed higher response rates with acceptable toxicities in breast, lung, and pancreatic cancers, suggesting that it could improve drug distribution in the body while inducing rapid elimination from serum (19–21). PM-Pac also had linear pharmacokinetics with lower peak concentration and shorter half-life than conventional paclitaxel, whereas the volume of distribution was greater after intravenous injection of PM-Pac (15), which can contribute to the removal of latent disseminated tumors not visible to the naked eye during debulking surgery.

To support these hypotheses, a recent phase III trial for comparing the effect between PM-Pac (230 mg/m^2^)/cisplatin and paclitaxel (175 mg/m^2^)/cisplatin has reported better tumor response and PFS in patients with lung cancer (12). Even though there is a lack of relevant phase III trials for ovarian cancer, two randomized controlled trials for evaluating hyperthermic intraperitoneal chemotherapy (HIPEC) have shown that HIPEC may improve survival in patients with advanced ovarian cancer who underwent ODS after neoadjuvant chemotherapy, suggesting that hidden peritoneal metastasis not resected during interval debulking surgery can be further removed by adding HIPEC (22, 23).

However, there were no differences in OS among the three cohorts. Considering that the effect of bevacizumab on improved OS was observed in only patients with high-risk factors for recurrence, including stage IV disease, inoperable stage III disease, and suboptimal debulking surgery in ICON7 study (24), cohort 3 consisted of a heterogenous population with various risk factors for recurrence, which failed to show the survival benefit of bevacizumab in this study. Furthermore, historical comparisons using retrospective cohorts 2 and 3, including the small number of patients, may act as a bias in interpreting that a survival benefit from PM-Pac can be expected as much as an improvement in PFS with the addition of bevacizumab.

Nevertheless, this study is meaningful in that it suggested for the first time the possibility that high-dose paclitaxel with a modified formulation can increase PFS, like adding bevacizumab to conventional chemotherapy in advanced ovarian cancer. Considering the trend of mixing targeted or immune oncologic agents with conventional chemotherapy to improve tumor response and survival in recent clinical trials such as the PAOLA-1 study (6), PM-Pac is expected to present the potential of showing a similar effect and reduced toxicities while decreasing the number of agents used together in advanced ovarian cancer. Moreover, the use of PM-Pac/carboplatin with short cycles can be considered to provide an equivalent or greater effect compared with conventional paclitaxel/carboplatin for treating early-stage ovarian cancer, with less toxicity due to reduced cycles, based on the results of this study. This hypothesis is also worthy of further evaluation in relevant clinical trials in the future.

## Data availability statement

The raw data supporting the conclusions of this article will be made available by the authors, without undue reservation.

## Ethics statement

The studies involving humans were approved by IRBs of Seoul National University Hospital (No. 1508-121-697) and Ajou University Medical Center (No.MED-OBS-15-321). The studies were conducted in accordance with the local legislation and institutional requirements. The participants provided their written informed consent to participate in this study.

## Author contributions

SP: Methodology, investigation, data curation, writing-original draft, visualization. J-HS: Software, validation, formal analysis, investigation, writing-review & editing. T-WK: Software, validation, formal analysis, investigation, writing-review & editing. S-JC: Conceptualization, investigation, resources, writing-review & editing, supervision. HK: Conceptualization, methodology, investigation, resources, writing-original draft, visualization, supervision. All authors contributed to the article and approved the submitted version.

## References

[1] McGuire WP Hoskins WJ Brady MF Kucera PR Partridge EE Look KY. Cyclophosphamide and cisplatin compared with paclitaxel and cisplatin in patients with stage III and stage IV ovarian cancer. N Engl J Med (1996) 334(1):1–6. doi: 10.1056/NEJM199601043340101 7494563

[2] Ozols RF Bundy BN Greer BE Fowler JM Clarke-Pearson D Burger RA. Phase III trial of carboplatin and paclitaxel compared with cisplatin and paclitaxel in patients with optimally resected stage III ovarian cancer: a Gynecologic Oncology Group study. J Clin Oncol (2003) 21(17):3194–200. doi: 10.1200/JCO.2003.02.153 12860964

[3] Moore K Colombo N Scambia G Kim BG Oaknin A Friedlander M. Maintenance olaparib in patients with newly diagnosed advanced ovarian cancer. N Engl J Med (2018) 379(26):2495–505. doi: 10.1056/NEJMoa1810858 30345884

[4] Gonzalez-Martin A Pothuri B Vergote I DePont Christensen R Graybill W Mirza MR. Niraparib in patients with newly diagnosed advanced ovarian cancer. N Engl J Med (2019) 381(25):2391–402. doi: 10.1056/NEJMoa1910962 31562799

[5] Burger RA Brady MF Bookman MA Fleming GF Monk BJ Huang H. Incorporation of bevacizumab in the primary treatment of ovarian cancer. N Engl J Med (2011) 365(26):2473–83. doi: 10.1056/NEJMoa1104390 22204724

[6] Ray-Coquard I Pautier P Pignata S Perol D Gonzalez-Martin A Berger R. Olaparib plus bevacizumab as first-line maintenance in ovarian cancer. N Engl J Med (2019) 381(25):2416–28. doi: 10.1056/NEJMoa1911361 31851799

[7] Mathew AE Mejillano MR Nath JP Himes RH Stella VJ. Synthesis and evaluation of some water-soluble prodrugs and derivatives of taxol with antitumor activity. J Med Chem (1992) 35(1):145–51. doi: 10.1021/jm00079a019 1346275

[8] Gelderblom H Verweij J Nooter K Sparreboom A. Cremophor EL: the drawbacks and advantages of vehicle selection for drug formulation. Eur J Cancer. (2001) 37(13):1590–8. doi: 10.1016/S0959-8049(01)00171-X 11527683

[9] Szebeni J Alving CR Savay S Barenholz Y Priev A Danino D. Formation of complement-activating particles in aqueous solutions of Taxol: possible role in hypersensitivity reactions. Int Immunopharmacol. (2001) 1(4):721–35. doi: 10.1016/S1567-5769(01)00006-6 11357884

[10] Sparreboom A van Tellingen O Nooijen WJ Beijnen JH. Nonlinear pharmacokinetics of paclitaxel in mice results from the pharmaceutical vehicle Cremophor EL. Cancer Res (1996) 56(9):2112–5. 8616858

[11] Sparreboom A van Zuylen L Brouwer E Loos WJ de Bruijn P Gelderblom H. Cremophor EL-mediated alteration of paclitaxel distribution in human blood: clinical pharmacokinetic implications. Cancer Res (1999) 59(7):1454–7. 10197613

[12] Shi M Gu A Tu H Huang C Wang H Yu Z. Comparing nanoparticle polymeric micellar paclitaxel and solvent-based paclitaxel as first-line treatment of advanced non-small-cell lung cancer: an open-label, randomized, multicenter, phase III trial. Ann Oncol (2021) 32(1):85–96. doi: 10.1016/j.annonc.2020.10.479 33130217

[13] Keam B Lee KW Lee SH Kim JS Kim JH Wu HG. A phase II study of genexol-PM and cisplatin as induction chemotherapy in locally advanced head and neck squamous cell carcinoma. Oncologist. (2019) 24(6):751–e231. doi: 10.1634/theoncologist.2019-0070 30796155 PMC6656523

[14] Kim HS Lee JY Lim SH Sun JM Lee SH Ahn JS. A prospective phase II study of cisplatin and cremophor EL-free paclitaxel (Genexol-PM) in patients with unresectable thymic epithelial tumors. J Thorac Oncol (2015) 10(12):1800–6. doi: 10.1097/JTO.0000000000000692 26484631

[15] Kim TY Kim DW Chung JY Shin SG Kim SC Heo DS. Phase I and pharmacokinetic study of Genexol-PM, a cremophor-free, polymeric micelle-formulated paclitaxel, in patients with advanced Malignancies. Clin Cancer Res (2004) 10(11):3708–16. doi: 10.1158/1078-0432.CCR-03-0655 15173077

[16] Lee SW Kim YM Kim YT Kang SB. An open-label, multicenter, phase I trial of a cremophor-free, polymeric micelle formulation of paclitaxel combined with carboplatin as a first-line treatment for advanced ovarian cancer: a Korean Gynecologic Oncology Group study (KGOG-3016). J Gynecol Oncol (2017) 28(3):e26. doi: 10.3802/jgo.2017.28.e26 28028994 PMC5391390

[17] Lee SW Kim YM Cho CH Kim YT Kim SM Hur SY. An open-label, randomized, parallel, phase II trial to evaluate the efficacy and safety of a cremophor-free polymeric micelle formulation of paclitaxel as first-line treatment for ovarian cancer: A korean gynecologic oncology group study (KGOG-3021). Cancer Res Treat (2018) 50(1):195–203. doi: 10.4143/crt.2016.376 28324920 PMC5784626

[18] Winer EP Berry DA Woolf S Duggan D Kornblith A Harris LN. Failure of higher-dose paclitaxel to improve outcome in patients with metastatic breast cancer: cancer and leukemia group B trial 9342. J Clin Oncol (2004) 22(11):2061–8. doi: 10.1200/JCO.2004.08.048 15169793

[19] Gradishar WJ Tjulandin S Davidson N Shaw H Desai N Bhar P. Phase III trial of nanoparticle albumin-bound paclitaxel compared with polyethylated castor oil-based paclitaxel in women with breast cancer. J Clin Oncol (2005) 23(31):7794–803. doi: 10.1200/JCO.2005.04.937 16172456

[20] Socinski MA Bondarenko I Karaseva NA Makhson AM Vynnychenko I Okamoto I. Weekly nab-paclitaxel in combination with carboplatin versus solvent-based paclitaxel plus carboplatin as first-line therapy in patients with advanced non-small-cell lung cancer: final results of a phase III trial. J Clin Oncol (2012) 30(17):2055–62. doi: 10.1200/JCO.2011.39.5848 22547591

[21] Von Hoff DD Ervin T Arena FP Chiorean EG Infante J Moore M. Increased survival in pancreatic cancer with nab-paclitaxel plus gemcitabine. N Engl J Med (2013) 369(18):1691–703. doi: 10.1056/NEJMoa1304369 PMC4631139 24131140

[22] Lim MC Chang SJ Park B Yoo HJ Yoo CW Nam BH. Survival after hyperthermic intraperitoneal chemotherapy and primary or interval cytoreductive surgery in ovarian cancer: A randomized clinical trial. JAMA Surg (2022) 157(5):374–83. doi: 10.1001/jamasurg.2022.0143 PMC8908225 35262624

[23] van Driel WJ Koole SN Sonke GS. Hyperthermic intraperitoneal chemotherapy in ovarian cancer. N Engl J Med (2018) 378(14):1363–4. doi: 10.1056/NEJMoa1708618 29617590

[24] Oza AM Cook AD Pfisterer J Embleton A Ledermann JA Pujade-Lauraine E. Standard chemotherapy with or without bevacizumab for women with newly diagnosed ovarian cancer (ICON7): overall survival results of a phase 3 randomised trial. Lancet Oncol (2015) 16(8):928–36. doi: 10.1016/S1470-2045(15)00086-8 PMC4648090 26115797

